# Lived experiences of everyday life during curative radiotherapy in patients with non-small-cell lung cancer: A phenomenological study

**DOI:** 10.3402/qhw.v10.29397

**Published:** 2015-11-24

**Authors:** Suzanne Petri, Connie B. Berthelsen

**Affiliations:** 1 Department of Oncology, Section for Radiotherapy, Rigshospitalet, Copenhagen University Hospital, Copenhagen, Denmark; 2 Section of Nursing, Institute of Health, Aarhus University, Aarhus, Denmark

**Keywords:** Hope, lived experiences, lung cancer, nursing, open qualitative interviews, phenomenology, radiotherapy, Reflective Lifeworld Research

## Abstract

**Aim:**

To explore and describe the essential meaning of lived experiences of the phenomenon: Everyday life during curative radiotherapy in patients with non-small-cell lung cancer (NSCLC).

**Background:**

Radiotherapy treatment in patients with NSCLC is associated with severe side effects such as fatigue, anxiety, and reduced quality of life. However, little is known about the patients’ experience of everyday life during the care trajectory.

**Design:**

This study takes a reflective lifeworld approach using an empirical application of phenomenological philosophy described by Dahlberg and colleagues.

**Method:**

A sample of three patients treated with curative radiotherapy for NSCLC was interviewed 3 weeks after the end of radiotherapy treatment about their experiences of everyday life during their treatment. Data were collected in 2014 and interviews and analysis were conducted within the descriptive phenomenological framework.

**Findings:**

The essential meaning structure of the phenomenon studied was described as “Hope for recovery serving as a compass in a changed everyday life,” which was a guide for the patients through the radiotherapy treatment to support their efforts in coping with side effects. The constituents of the structure were: Radiotherapy as a life priority, A struggle for acceptance of an altered everyday life, Interpersonal relationships for better or worse, and Meeting the health care system.

**Conclusion:**

The meaning of hope was essential during radiotherapy treatment and our results suggest that interpersonal relationships can be a prerequisite to the experience of hope. “Hope for recovery serving as a compass in a changed everyday life,” furthermore identifies the essentials in the patients’ assertive approach to believing in recovery and thereby enabling hope in a serious situation.

Lung cancer is the second most frequent type of cancer in Denmark and approximately 4500 people are diagnosed annually (Cancerregistret, 2012). Lung cancer is divided into small cell lung cancer and non-small-cell lung cancer (NSCLC); up to 90% of cancer cases are caused by smoking. NSCLC accounts for approximately 83% of all cases and is treated with surgery, chemotherapy, radiotherapy, or a combination, depending on the stage of disease. Surgery and chemotherapy has been the most effective choice of treatment for NSCLC. Within the last 10–15 years, radiotherapy has shown promising results as curative treatment for lung cancer (Hansen, Paarup, Sørensen, Hansen, & Werenberg, 2005) delivered once a day during a period of 6 weeks.

Patients with NSCLC try to maintain their everyday life during radiotherapy treatment; however, this is often perceived as difficult because of the overwhelming insecurity about their future prospects (Ekfors & Petersson, 2004). Feeling stigmatized and guilty about the possibility of having caused the disease themselves due to long-term smoking can compromise the effort to maintain a normal everyday life (Chapple, Ziebland, & McPherson, 2004; Hansen & Savatsky, 2008). The severe side effects of radiotherapy treatment such as coughing, dyspnoea, pain, and fatigue also have an impact on the lives of the patients (Ekfors & Petersson, 2004). In a longitudinal qualitative study of factors that influence the distress of patients with lung cancer, Lowe and Molassiotis (2011) found that symptoms well known to the patients were easier to cope with than new and unfamiliar symptoms. The patients reduced their level of distress by connecting their symptoms to socially acceptable actions instead of the disease; thus, fatigue was caused by doing housework and not by the disease (Lowe & Molassiotis, 2011).

Other studies have shown how increased knowledge and ability to cope with symptoms through nursing interventions can have a positive effect on the quality of life of patients with lung cancer during radiotherapy treatment (Chan, Richardson, & Richardson, 2011; Clark et al., 2013). In a descriptive longitudinal study of 23 patients with NSCLC receiving curative radiotherapy, John (2001) found that quality of life was significantly lower during and 1 month after radiotherapy treatment and increased again 4 months after treatment. Langendijk and colleagues (2001) found similar results in their prospective study of quality of life in 164 patients during radiotherapy. However, they also identified an increase in emotional functioning during radiotherapy; this was correlated to an increasing hope for the future (Langendijk et al., 2001), often seen in cancer patients during active treatment.

Studies have shown how quality of life of patients with NSCLC may decrease during radiotherapy treatment (John, 2001; Langendijk et al., 2001) but also how nursing interventions during the treatment can have a positive impact on quality of life (Chan et al., 2011; Clark et al., 2013). The patients experienced that maintenance of a normal everyday life during treatment was important but difficult (Ekfors & Petersson, 2004). However, little is known about how patients’ everyday life is actually lived and experienced.

## Aim

The aim of the study was to explore and describe the essential meaning of the phenomenon: Everyday life during curative radiotherapy in patients with NSCLC.

## Methods

The study was conducted within the descriptive phenomenological approach of *Reflective Lifeworld Research* (Dahlberg, Dahlberg, & Nyström, 2008). Dahlberg and colleagues developed the reflective lifeworld approach as a research method with epistemological and ontological roots in the philosophy of phenomenology by Husserl and Merleau-Ponty (Dahlberg et al., 2008). The philosophical inspiration is seen in the focus on explaining the complexities of the lived experiences. In the lifeworld humans take a natural stance without consciously reflecting on their actions or experiences (Dahlberg et al., 2008). The aim of reflective lifeworld research is to describe the studied phenomenon as it is experienced by the participants. With this approach the researchers try to uncover the phenomenon while staying open to the participants’ expressed meaning of their lifeworld (Dahlberg et al., 2008).

Openness is an essential concept in phenomenological philosophy (Dahlberg et al., 2008). The importance of staying open as a researcher and holding back your pre-understanding of the phenomenon is therefore an important ability introduced by Husserl as *bracketing* (Husserl, 2000/1928). However Dahlberg and colleagues (Dahlberg et al., 2008) introduce the term *bridling* instead of *bracketing*, which encompasses the *bracketing* of pre-understanding but even more essential the patience and *holding back* the understanding through the research process through the act of dwelling with the phenomenon. As Dahlberg (2006) argues, “… we bridle the understanding so that we do not understand too quickly, too carelessly, or slovenly, or in other words, that we do not make definite what is indefinite” (p. 16).

To embrace the concept of *bridling* we took a critical reflective stance initially and throughout the study towards our pre-understanding of the studied phenomenon: Everyday life during curative radiotherapy in patients with NSCLC. Our pre-understanding is that patients with NSCLC receiving curative radiotherapy are not only struggling in an everyday life with symptoms and side effects of the treatment but also with feelings of guilt, shame, and stigmatization. We further believe that the needs of this patient group are overlooked in nursing as well as in the progress of standardized care and treatment. During the phases of the study we continuously and patiently reflected on and discussed the phenomenon and its embedded meanings to secure an open and bridled attitude, especially when our pre-understanding was reflected in the meanings.

### Participants and setting

The participants in the study were recruited from a department of oncology at a Danish university hospital by the first author. Eligible participants were identified through the department's electronic booking system by the first author, who had no prior knowledge about the patients. The participants were then approached by the first author and invited to participate in the study in the department's waiting area immediately after their last radiotherapy treatment. Four patients were identified as eligible in the study period, and they were invited and consented to participate. One withdrew before the interview due to severe side effects of the treatment.

Three participants, one woman and two men between 65 and 72 years of age, were thus included in the study. All participants had been treated with radiotherapy with curative intent for NSCLC. Two participants were retired whereas one was on sick leave. Inclusion criteria were completion of radiotherapy treatment within the last 2–3 weeks at the time of the interview and ability to speak and understand Danish. Furthermore, the participants should have lived in their own home during the radiotherapy treatment to ensure the perspective of everyday life (Jacobsen, 2009).

We refrained from conducting more than the three interviews to avoid disturbing more patients unnecessarily in their vulnerable situation and to protect the moral sensitivity to the patients’ values in these situations (Heggestad, Nortvedt, & Slettebø, 2013; Nordic Nurses’ Federation 2003).

### Data collection

The first author conducted open qualitative interviews in spring 2014. During the interviews the first author focused on the explored phenomenon: Everyday life during curative radiotherapy in patients with NSCLC and its meaning in accordance with the reflective lifeworld approach (Dahlberg et al., 2008). The interviewer encouraged participants to elaborate on different experiences of everyday life during the care trajectory guided by the lifeworld fragments, inspired by Merleau-Ponty: Selfhood, sociality, embodiment, temporality, spatiality, project, and discourse (Ashworth, 2003). The interviewer was constantly aware of not taking any meaning for granted to sustain an open and bridled attitude (Dahlberg et al., 2008). An interview guide was thus not used during the interviews to ensure the open and bridled attitude (Dahlberg et al., 2008).The interviews began with an opening question: “How is a typical day for you?” This question was followed by the question: “How was a typical day for you during the radiotherapy treatment?” The interviews were conducted in the participants’ homes and lasted from 48 to 66 minutes. The interviews were digitally recorded and subsequently transcribed verbatim by the first author to ensure inclusion of non-verbal language by the participants during the open interviews. Following the first interview we discussed and critically reflected on the immediacy and openness in the first interview, and subsequently, the interviewer used the reflections to strive for an open and *bridled* attitude during the next interviews.

We only conducted one interview per participant, because our main focus was on the unreflective experiences during the treatment trajectory. The risk of getting too many reflected experiences from the participants refrained us from repeating the interviews (Dahlberg et al., 2008).

### Data analysis

Data analysis in reflective lifeworld research is similar to other descriptive phenomenological methods (Dahlberg et al., 2008). A descriptive phenomenological analysis is based exclusively on the collected data and the aim is to describe the phenomenon without seeking interpretation or explanation and to illuminate the essence of the phenomenon. Through the phenomenological analysis the essential meaning structure and its constituents are revealed and described at length (Dahlberg et al., 2008).

During the data analysis we searched for the essential meaning of the phenomenon: Everyday life during curative radiotherapy in patients with NSCLC. The analysis in reflective lifeworld research is based upon the “hermeneutic rule” of Gadamer and performed in an alternation between the whole and the parts (Dahlberg et al., 2008). This analysis included three phases and it was essential to preserve an open phenomenological attitude to the data throughout every phase. We have illustrated the process of analysis in Table I with specific emphasis on the constituent *a struggle for acceptance of an altered everyday life*.

**Table I:** The three phases of the data analysis.

1. Phase Reading and rereading of transcripts		2. Phase Grouping of meaning units into clusters	3. Phase Assembling the clusters of meaning in patterns
	
Excerpt from interview	Content	Meaning unit	Constituent
“… my body said stop, I felt that … so I told myself that I could just do it tomorrow …”	The side effects of the radiotherapy treatment are so disabling that the body *says stop* and the participant accepts that	Acceptance of a limited physical ability	A struggle for acceptance of an altered everyday life
“… yes it has been tough, because I haven't had the energy to do anything else than radiotherapy …”	The participant acknowledges that he only has energy to get the treatment		

In the first phase the transcribed interviews were read and reread until we were able to divide the text into meaning units related to the phenomenon. During this phase we noticed how the participants continuously stated how the treatment was limiting their everyday life. The second phase included grouping of meaning units across data where participants expressed differentiated physical limitations in their everyday life; however, the common denominator was the ongoing search for acceptance of the limitations in everyday life. This iterative process of grouping and regrouping into clusters of meaning continued until, through reflection and discussion, we were in agreement on the right clusters, continuing the process of analysis to finding the constituent *a struggle for acceptance of an altered everyday life*. The third and final phase of the analysis was assembling the clusters of meaning into patterns that constituted the phenomenon studied.

Throughout the analysis we constantly reflected and discussed upon alternative meanings to a given meaning unit partly by relating the meaning unit to the other meaning units and the data set as a whole. This analytical process was performed by simultaneously rereading the interview transcripts several times to ensure that the meaning units were consistent with the data as a whole. During this iterative, persistent, and circular process a pattern in the meaning clusters appeared and subsequently the essential meaning structure and constituents of the phenomenon emerged.

### Ethical considerations

In accordance with Danish legislation, this study did not need approval by an ethics committee. Written and oral information was given to the participants, and written consent was obtained according to ethical guidelines for nursing research in the Nordic countries (Nordic Nurses’ Federation 2003). The interview data were processed and stored according to rules by the Danish Data Protection Agency (2014).

We chose to interview the participants after the treatment trajectory to protect physically and emotionally burdened individuals (Larsson, Hedelin, & Athlin, 2007; Röing, Hirsch, & Holmström, 2007). The interviews were conducted 3 weeks after completion of the radiotherapy treatment to ensure that the participants had recovered from the early side effects of the treatment and before possible long-term side effects were manifested (Degerfält, Moegelin, & Sharp, 2008; Stone, Coleman, Anscher, & McBride, 2003); moreover, the participants still remembered the experience of everyday life during radiotherapy treatment.

## Findings

The essential meaning structure of the phenomenon studied, everyday life during curative radiotherapy for patients with NSCLC, is characterized as *hope for recovery serving as a compass through the changed everyday life*. Hope for recovery permeates everyday life and everything evolves around this hope. Simultaneously hope for recovery becomes a guiding light and thereby enables the patients to stay on track during the turbulent everyday life.

In order to navigate in everyday life, the daily encounters with and the support from the health care system are crucial. Embedded in the encounter is the importance of the close cooperation with and acknowledgement from the health care professionals. The patients experience an everyday life that is temporarily put on hold, while the challenging task of completing the radiotherapy treatment trajectory is the only priority in everyday life. A quest and struggle for acceptance of limited physical and social abilities pervades everyday life and as a consequence structure and predictability becomes necessary in order to navigate in everyday life. The patients also experience a need for closeness and intimacy as well as a need for distance and disengagement in their interpersonal relationships to pull through the treatment trajectory.

**Figure 1:**
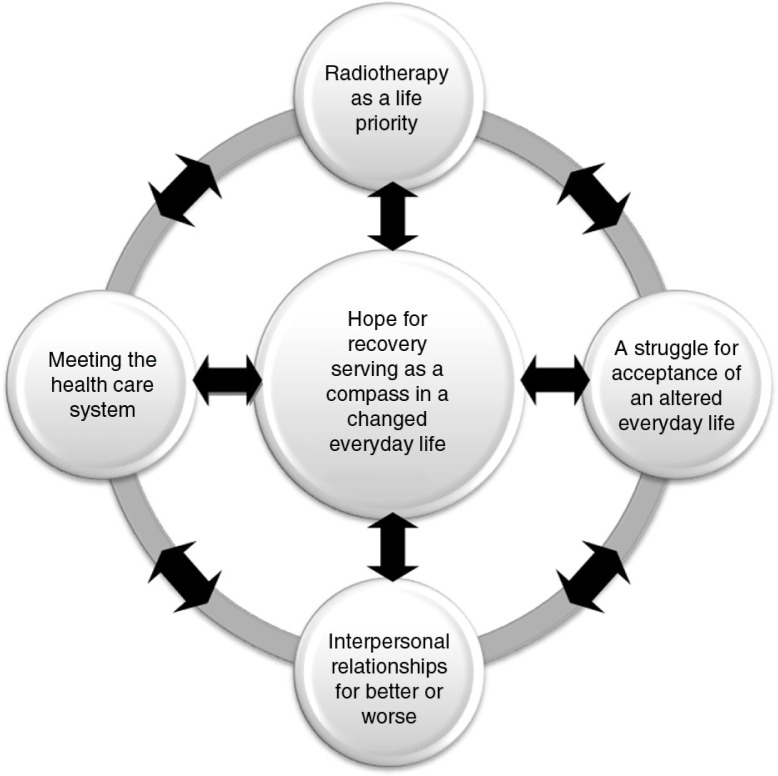
The essential meaning structure of hope for recovery serving as a compass through the changed everyday life and the four constituents.

The essential meaning structure of the phenomenon is further enlightened by its four constituents (Figure 1): Radiotherapy as a life priority; A struggle for acceptance of an altered everyday life; Interpersonal relationships for better or worse; and Meeting the health care system.

The four constituents are closely intertwined and are all prerequisites of each other. The understanding of an individual constituent is extended through the descriptions of the others and is an expression of the whole and parts of the whole, as emphasized by Dahlberg and colleagues (2008).

### Radiotherapy as a life priority

This constituent was identified through the participants’ persistent focus on managing and coping with the treatment. The participants had to prioritize the treatment in their everyday lives and they channelled their limited physical and mental resources into this priority, as reflected in the following passage:

Life is immediately put on hold … so a normal everyday life didn't concern me because everything evolved around treatment and only completion of the treatment was important, so everything else didn't matter. (P1)

Consequently, the remains of their everyday lives became a secondary priority to completing the treatment, similar to the participants’ past and present work life. Completing the treatment was through this comparison experienced as a great achievement by the participants and made the radiotherapy more tolerable; it was like a job that had to be done. The participants explained the vacuum they experienced after completing the treatment:

Now I have lived for something, to complete and survive the treatment, and suddenly the priority of life is gone. (P1) and

It's kind of like being in a limbo, you don't know if you are cured or not. You just have to wait. (P2)

To navigate in everyday life during radiotherapy, hope for recovery was important. *Hope for recovery* served as a compass because the life priority was difficult to focus on due to the turbulent everyday life with daily treatments and severe side effects such as fatigue, pain, nausea, vomiting, and dyspnoea. The participants fought to keep up their spirits by focusing on the completion of the treatment, the life priority, despite the awareness of the poor prognosis; and the hope for recovery was perceived as a guiding light in everyday life. One participant reflected:

But in 20% of the cases the cancer is cured … and this is what you should focus on … the statistics will never be good. (P1)

Another participant said:

Well, I am convinced that I will be cured; I believe that, maybe I will have some detours … but cured, I do believe it. (P2)

The meaning of the latter quotation shows the importance of genuine belief in recovery to navigate through the changed everyday life.

### A struggle for acceptance of an altered everyday life

To try to accept the altered everyday life during radiotherapy treatment became a persistent struggle for the participants. Meanwhile they repeatedly experienced how their normal everyday life had changed and this called for a change of mindset. An everyday life with a diversity of activities was reduced because of the side effects of the treatment. The participants’ everyday lives had previously consisted of social, physical, intellectual, and cultural activities but their lifeworlds were now limited by the treatment. This aspect changed the importance of activities to the participants and therefore activities such as shopping and daily household chores were minimized. Simultaneously, the struggle for acceptance of an altered everyday life was affected by the urge to feel better and maintain a normal everyday life. Being confronted with other patients’ easy way through radiotherapy made this desire even more evident:

Yes it has been tough, because I haven't had the energy to do anything else than radiotherapy … those [other patients seen in the waiting area] who just come in, get the treatment and are off to work, it must be damn great. (P2)

The participants now searched for meaning in other everyday activities than previously and attached new meaning to activities that used to be less important. This could be reading, watching television, listening to the radio, or other sedentary activities that corresponded to what was physically possible to the individual:

I just lay in my bed; I couldn't drive to the hospital, so my friends had to accompany me. And I just went to bed again when I came home … eventually I didn't eat, I didn't drink, I did nothing. I just stayed in bed and waited for the next day to come so I could get my treatment … well, it was like hell. (P1)

Through the struggle for acceptance the participants experienced that they were able to navigate in the altered everyday life, even when it sometimes felt like *hell*. Another important issue in the struggle for acceptance was the acknowledgement of the necessity of asking for help; such as getting rides from friends to the hospital or letting your daughters do the grocery shopping.

An additional perspective was the importance of structure and predictability in the altered everyday life. The participants assigned importance to being in control of the care trajectory. It was important to have approximately the same appointment time every day because this gave participants a feeling of structure and predictability, and consequently a sense of control in an otherwise uncontrollable situation:

I need to have such commitments [the radiotherapy treatment] done, and then I feel okay and have the rest of the day to myself … I cannot cope with things I haven't anticipated, I need time to adjust. (P3)

Through this sense of control it was possible to navigate through the limitations that characterized the altered everyday life.

### Interpersonal relationships for better or worse

The importance and meaning of relationships with other people got a new dual perspective during the treatment trajectory. Intimacy in interpersonal relationships was important to the participants and they experienced how the altered everyday life changed the nature of their interpersonal relationships. The participants felt cared for to a much greater extent than previously, through daily telephone conversations and frequent visits from close relatives and friends. Thus, in some situations the altered everyday life nourished the interpersonal relationships and strengthened the network in general:

Yes they [family and friends] have become much more caring and visit me in a completely different way … so we have grown much closer …. Now we're talking every day and then you talk also about … well about the snowdrops in the garden and they have really grown and look much better today than yesterday … I would not have spent so much time talking about such small things before. (P1)

Opposing that, the participants experienced that the interpersonal relationships could challenge everyday life. A need for distance occurred due to insufficient nutritional intake caused by severe side effects. The pressure from family and friends to eat more became too overwhelming resulting in lies and dishonesty to avoid pain and discomfort:

I had no recurring person [family and friends] … they couldn't really keep up with me so I cheated; I said that I had eaten, but it was a lie. (P1)

The need for distance and disengagement in interpersonal relationships became clear through social activities, too. The participants experienced discomfort when they took part in social encounters where the cancer and the treatment were not explicated and everyone just pretended that everything was fine. Simultaneously the participants found it difficult to talk about the cancer and treatment, consequently creating a distance in interpersonal relationships.

### Meeting the health care system

The daily encounters with the health care professionals in the radiotherapy department during the treatment trajectory had significant value to the participants. They experienced that the cooperation and interaction had a positive impact on their everyday lives. A substantial part of this interaction was to feel accepted and taken seriously, experiencing comfort and thus enabling the participants to manage the side effects of the radiotherapy:

The understanding and the feeling of not being a burden, but they take it seriously … they are so kind and you do not feel like a drag at all. (P1)

The experience of not being a burden became very important to navigate in everyday life, and the relationship with the health care professionals created a sense of comfort and ease in the technology-intensive context. Participants experienced that the radiation therapists mastered the technical part of the treatment, while they simultaneously appreciated that the relationship was characterized by mutual respect, genuine interest, and humour:

I felt safe, I felt very comfortable in the relationship … and several of them [radiation therapists] also had a spark of humour. (P3)

However, the physical environment of the health care system had an impact on the experience too. The participants emphasized that the radiotherapy department was perceived as a non-hospital-like environment, which made them feel more like individuals and less like patients. To be involved in planning the treatment appointment schedule was meaningful too. The participants obtained structure and predictability in the altered everyday life by using this influence, resulting in a sense of control. The experience of influence was closely linked to the experience of acknowledgement. Through the encounters with health care professionals the participants felt acknowledged as individuals and not just as patients with a cancer diagnosis.

Another perspective of the encounter had a negative impact on everyday life. The legal obligation from the health care professionals on giving thorough information on diagnosis, treatment, and prognosis while obtaining informed consent to the treatment, could threaten the participants’ experience of hope:

They made a mistake by emphasizing on the statistics, and statistics suck … and that is what you remember from the information … that you have no future. (P1)

Hope for recovery and confidence in getting well were key factors to navigate in everyday life and the participants experienced the thoroughness of the information as useless and on the verge of being detrimental. Thus, the health care system played a dual role in relation to hope; partly by offering hope through treatment and care, and partly by threatening hope through provision of information in the process of informed consent.

## Discussion

In our study we found that hope for recovery was essential to navigate in everyday life. We suggest that this hope can be viewed as the means that made it possible for the participants to complete the radiotherapy treatment. The participants in our study experienced a distinct hope and belief in recovery during the treatment trajectory and this hope made it possible to navigate in everyday life.

Existing empirical studies of patients with lung cancer undergoing radiotherapy treatment have also identified the importance of hope (Ekfors & Petersson, 2004; Gamble, 1998). Similar to our results, in a Swedish qualitative study of 15 patients with lung cancer interviewed during radiotherapy treatment, Ekfors and Petersson (2004) found that hope and belief in recovery was necessary to endure treatment. We found that the participants had to put their lives on stand-by and make the radiotherapy treatment a life priority to complete it and thereby retain hope. This also corresponds to a grounded theory study by Gamble (1998) including 15 patients with cancer among whom 6 had lung cancer, which suggests that full focus on the radiotherapy treatment instils and promotes the experience of hope in patients with lung cancer. The dimension of hope in everyday life can be further understood by drawing on the philosophical work of Marcel (1951, 1956, 1967, 2010). Marcel suggests that hope can be seen as concrete, like a desire for something, but more importantly, as a universal hope, like a basic feeling that can empower the individual to cope in and with a difficult situation (1956, 1967). In our study hope took both forms, partly by being concrete as in *hope for recovery*, the means that made it possible for the participants to complete the radiotherapy treatment; and partly by being more universal as *a compass to navigate in everyday life* – a guiding light that kept the participants on the right track in the turbulent everyday life. Another important finding in our study was the participants’ firm and assertive beliefs in recovery and their will to complete the radiotherapy treatment, although they were fully aware of the gloomy statistics in relation to prognosis. Marcel's (1956, 1967) notions of the transcending hope can explain this assertiveness and willpower. The essence of hope has the possibility of transcending empirical evidence, and thus hope can be present despite illness and poor prognosis (Marcel, 1956, 1967). Therefore this is an important point to be considered by the health care professionals when having conversations on prognosis with these patients, because it is essential to experience hope to endure the toxic treatment.

The participants in our study accepted that their everyday lives had been altered due to the treatment and its side effects, and the *struggle for acceptance* was necessary in order to endure and complete the treatment, as it had become a life priority. This result was supported by another study of patients with lung cancer, which suggested that a normal everyday life is impossible during radiotherapy treatment (Lowe & Molassiotis, 2011). Contrasting these findings are the results from the study by Ekfors and Petersson (2004) where the participants were able to maintain a normal everyday life during radiotherapy treatment. A possible explanation of this difference could be the timing of the interviews; in the Swedish study the participants were interviewed in the second week of the radiotherapy treatment before the early side effects of the treatment had manifested themselves (Degerfält et al., 2008) and therefore they were still able to maintain a normal everyday life.

In our study we identified the importance and the dialectics of relationships through the constituent *interpersonal relationships for better or worse*, where both withdrawal from social situations and intimacy in relationships were necessary. This is in accordance with the results by Ekfors and Petersson (2004), who found that social support was a prerequisite to endure the radiotherapy treatment and the side effects. Simultaneously the participants in our study felt a need to withdraw from social situations, which also was identified by Ekfors and Petersson (2004). Their results suggested that this withdrawal could result in social isolation (Ekfors & Petersson, 2004), which was contrary to our findings where none of the participants felt socially isolated. In our study the participants were so affected by the side effects that they could not manage everyday life on their own, and had to ask for practical help and relational support. The importance of interpersonal relationships can also be identified in Marcel's (1951, 1967, 2010) notions on hope. Marcel suggests that hope rises from a communion, through a mutual relationship amongst human beings and thus we argue as Marcel (1951, 1967, 2010) that interpersonal relationships are a prerequisite to the experience of hope and interpersonal relations nourish hope.

The meeting and cooperation with the health care professionals was also an important factor in everyday life. The participants experienced that it was important to feel acknowledged, taken seriously and met as individuals, which was in line with the results of the study by Gamble (1998). The importance of not being seen as a burden in the eyes of the health care professionals was essential in our study as in the study by Gamble (1998). The significance of gaining influence and having a sense of control in everyday life whilst feeling safe in the technology-intensive context corresponds to the results of the Swedish study, where the participants appreciated the influence and cooperation with competent doctors and nurses (Ekfors & Petersson, 2004). Our results also suggest that the radiation therapists, who interact with the patients on a daily basis, have to be aware of the influence they have on the patients’ experiences during the treatment trajectory. The results from a Norwegian phenomenological study by Egestad (2013), of 11 patients with head and neck cancer in radiotherapy, confirms and extends our results by suggesting that contact with radiation therapists can lead to increases or decreases in uncertainty and existential anxiety. Likewise the importance of feeling safe and secure in the high-tech environment, which we identified, had great significance in the study by Egestad (2013).

### Methodological considerations

Only three participants were included in our study, and caution should be taken to generalize results. However, according to Dahlberg et al. (2008) any experience in a phenomenological study is important regardless of the number of participants experiencing it. Dahlberg and colleagues (2008) defends the problem of few participants and describes how the depth and intensity of the description is more important than the number of participants. In our study we obtained a wide variation of experiences of everyday life despite the small number of participants, but we recognize the limitations in data amount and quality that could be implied in the few participants. Still the results of a phenomenological study will always be contextual and can be extended and nuanced indefinitely by including more participants (Dahlberg et al., 2008).

The recruitment of participants was affected by circumstances beyond our control with few eligible participants in the recruitment period and an increase in patients not completing the radiotherapy treatment due to severe side effects.

The first author, who is a novice researcher, conducted the open interviews and this could have impaired the quality and richness of the interview data. However, we continuously used critical reflection and awareness through the research process to ensure a high level of openness as requested in phenomenological studies and thereby tried to compensate for possible impairments (Dahlberg et al., 2008). Furthermore none of the participants expressed issues with guilt, shame, and stigmatization, as our pre-understanding entailed, which suggest that openness prevailed in the study.

The study was conducted in Denmark and the impact of the daily encounter and cooperation with the health care professionals were important findings; therefore, the organisational structures of the health care system must be considered when transferring the results. We suggest that the results can be transferred to countries with similar health care systems as the Danish, e.g., the Nordic countries (Magnussen, Vrangbæk, Saltman, & Martinussen, 2009).

Despite the methodological limitations we still argue that our findings extend and complement the limited existing knowledge and contribute with novel and valuable insights into the phenomenon under study, however further research on the phenomenon is warranted.

## Conclusion and implications for practice

This study highlights the struggle patients with NSCLC experience during radiotherapy treatment with curative intent. The side effects of the treatment change the patients’ everyday lives tremendously, and the treatment becomes overshadowing in everyday life. Our study indicates that interpersonal relationships are a prerequisite of experiencing hope. Additionally, *hope for recovery serving as a compass in a changed everyday life* identifies the essentials in the patients’ assertive approach to believe in recovery and thereby enabling hope in a serious situation. The meaning and importance of hope was essential in our study and the findings suggest that hope permeates the experience of everyday life, and that the feeling of hope makes patients able to complete radiotherapy treatment.

Our findings provide new insights into the lives of these vulnerable patients and give health care professionals new knowledge that can inform and guide them to provide evidence-based care. Since interpersonal relationships are essential in everyday life and a prerequisite to experiencing hope, we suggest that the care could be directed at encouraging and supporting the patients in formation and maintenance of interpersonal relationships. Interventions could advantageously be directed at patients with few interpersonal relationships through closer follow-up during the treatment trajectory, thus through the relations with the health care professionals hope could be nourished. Another important implication for practice is the acknowledgement of hope as an individual experience that enables the patients to endure the treatment, and consequently has to be respected no matter how unrealistic it may seem to the health care professionals.
